# Data set on effect of amaranth proteins on the RAS system. *In vitro*, *in vivo* and *ex vivo* assays

**DOI:** 10.1016/j.dib.2020.105168

**Published:** 2020-01-23

**Authors:** Santiago Suárez, Paula Aphalo, Gustavo Rinaldi, Alejandra Quiroga, María Cristina Añón

**Affiliations:** a Centro de Investigación y Desarrollo en Criotecnología de Alimentos (CIDCA-CONICET-CIC-UNLP), Universidad Nacional de La Plata, La Plata, 1900, Argentina; b Facultad de Ciencias Médicas, Universidad Nacional de La Plata, La Plata, 1900, Argentina

**Keywords:** Amaranth proteins, Protein hydrolysates, Bioactive peptides, Renin-angiotensin system, Antihypertensive emulsions, Spontaneously hypertensive rats

## Abstract

Data set presented in this article is related to the research paper entitled “Effect of amaranth proteins on the RAS system. *In vitro, in vivo* and *ex vivo* assays”, available in Food Chemistry [1]. In this article, we evaluated the effect on systolic blood pressure of spontaneously hypertensive rats (SHR) of different samples with amaranth proteins/peptides. The effect of these samples on RAS system was evaluated using *in vitro* and *ex vivo* assays. The concentration of renin and angiotensin converting enzyme (ACE) was evaluated using two commercial ELISA kits. Renin concentration was estimated through a direct immunoassay and ACE concentration with an immunoassay based on a competitive inhibition. In addition, the ACE inhibitory activity in plasma was evaluated using a spectrophotometric assay according to [2]. *Ex vivo* experiments were done with thoracic aorta extracted during the surgical procedure employed to obtain blood samples according to [3]. Data presented in this article recollect a very extensive work on how can be affect the RAS system in SHR model using amaranth protein/peptides as potential antihypertensive samples. These data could be useful to design novel functional foods for hypertensive individuals.

**Table:** Specifications Table

Subject	Food Science
Specific subject area	Bioactive peptides Antihypertensive peptides
Type of data	Table
How data were acquired	A tail cuff and a pulse sensor (NarcoBiosystems. Houston. TX) were used for recording the systolic blood pressure. ELISA test for ACE and renin plasma concentration. Spectrophotometric assay for ACE activity. Contractile response of aortic smooth muscle was measured in a force transducer (Grass FT.03D. Grass Telefactor. West Warwick. CT. USA).
Data format	Raw
Parameters for data collection	Sixty male SHR rats were used weighed between 210 - 290 g and had approximately 10 weeks of age at the beginning of the study. Rats were housed in stainless steel cages. 4 animals per cage, with sterilized bedding. The facility had air conditioning and a 12 h light −12 h dark cycle. Food and water were provided *ad libitum*. Tap water was provided in sterilized bottles with stainless steel nipples. Animals were fed with extruded balanced feed.
Description of data collection	Animals were divided into 8 groups of 7–8 animals each, except captopril and aliskirene groups in which 4 animals were used. Samples were administered by the orogastric route with 2 ml of each sample dispersed in distilled water. The systolic blood pressure was measured according to [3]. After surgical procedure the rat's abdominal aorta was then cannulated to collect a blood sample (roughly 6 ml) in heparin-coated tubes. The plasma was separated and used for plasma ACE. Renin concentrations and plasma ACE activity. Also, the thoracic aorta was resected and then cut into two mm long rings. The *ex vivo* assay was performed according to [3].
Data source location	Institution: CIDCA. UNLP. CONICET City/Town/Region: La Plata. Buenos Aires Country: Argentina
Data accessibility	With the article
Related research article	Author's name: Santiago Suárez. Paula Aphalo. Gustavo Rinaldi. María Cristina Añón. Alejandra Quiroga Title: Effect of amaranth proteins on the RAS system. In vitro. in vivo and *ex vivo* assays Journal: Food Chemistry DOI: 10.1016/j.foodchem.2019.125601

**Table:** 

**Value of the Data** • This data collects *in vitro, in vivo* and *ex vivo* information about amaranth protein and peptides with antihypertensive effect. The interaction of the different approaches is important for understanding the mechanism of action of amaranth peptides. • The data will help to understand the possible mechanism of action of food peptides on the RAS system. • Data are useful for researchers and academician to acquire innovative knowledge about the effect of bioactive peptides on RAS system. In addition, data provide new insights and information to consider in the design of novel functional foods with amaranth what could be useful for entrepreneurs and food industry. • How affect the bioactive peptides on RAS system is a valuable tool to develop a new functional food. Emulsions with amaranth proteins could be a delivery system of antihypertensive peptides with the possibility to enhance the biodisponibility and reach the target organ successfully.

## 1 Data description

Data describes the effect of amaranth protein/peptides on RAS system inquiring into the mechanism of action of these samples using *in vitro*, *in vivo* and *ex vivo* approaches [1].

Treatment groups were:

1. G_W_: Negative control group. Animals treated with water, which did not receive amaranth proteins.
2. G_C_: Captopril group. Animals treated with captopril, an ACE inhibitor.
3. G_A_: Aliskiren group. Animals treated with aliskiren, a renin inhibitor.
4. G_API_: API group. Animals treated with amaranth protein isolated (API).
5. G_AH_: AH group. Animals treated with amaranth protein hydrolysate (AH).
6. G_VIKP_: VIKP group. Animals treated with the synthetic peptide VIKP.
7. G_E_: w/o Emulsion group. Animals treated with w/o emulsion.
8. G_E+VIKP_: w/o Emulsion + VIKP group. Animals treated with w/o emulsion added with VIKP.

In order to compare mean values, a one way analysis of variance (ANOVA) multiple comparisons was applied. The critical significance level was set at p < 0.05. All samples were compared to G_W_ (negative control group).

Table 1 shows the reduction in SBP values exerted in each experimental group. Data were expressed as the decrease of SBP in mmHg of animals 3 h after the administration of each sample with respect to the SBP measured at the beginning of the experiment (SBP_3h_-SBP_0hi_). ΔP values are presented as mean ± SEM. Animals belonging to the G_E_ and G_E+VIKP_ groups showed the most significant reduction in the SBP reaching reduction values of 42 ± 2 mmHg and 35 ± 2 mmHg respectively. The administration of API, AH or VIKP in water as vehicle (G_API_, G_AH_ y G_VIKP_ groups) caused a reduction in SBP values that was significantly lower than those observed in the groups mentioned above (25 ± 14 mmHg, 26 ± 3 mmHg and 21 ± 3 mmHg respectively.)

**Table 1:** Systolic blood pressure before and after treatment. ΔP (SBP_3h_-SBP_0hi_) values are presented as mean ± SEM.

Treatment group		SBP (mm Hg)													
		1	2	3	4	5	6	7	8	9	10	11	12	13	14
G_W_		196	281	215	190	188	206								
		176	280	193	195	177	196								
		200	273	244	202	170	201								
	average	191	278	217	196	178	201								
	post intragastric administration														
		201	314	208	228	167	188								
		186	283	179	222	152	187								
		196	244	156	203	162	250								
	average	194	280	181	218	160	208								
	ΔP = SBP3_h_-SBP_0hi_	4	2	−36	22	−18	7								
	**ΔP average**	**-3**^**a**^													
	**SEM**	**8**													
G_A_		196	185	195	196										
		197	183	187	192										
		190	186	185	193										
	average	194	185	189	194										
	post intragastric administration														
		145	156	139	139										
		147	154	140	135										
		152	150	145	140										
	average	148	153	141	138										
	ΔP = SBP3_h_-SBP_0h_	−46	−31	−48	−56										
	**ΔP average**	**−45**^**b**^													
	**SEM**	**5**													
G_C_		208	205	–	205										
		199	205	–	193										
		199	205	–	183										
	average	202	205		194										
	post intragastric administration														
		166	171	–	168										
		159	173	–	171										
		162	177	–	163										
	average	162	174		167										
	ΔP = SBP3_h_-SBP_0h_	−40	−31		−26										
	**ΔP average**	**−32**^**b**^													
	**SEM**	**4**													
G_API_		166	162	197	180	259									
		160	166	208	187	238									
		159	168	204	190	234									
	average	162	165	203	186	244									
	post intragastric administration														
		153	155	136	140	179									
		145	157	147	154	173									
		158	154	152	162	182									
	average	152	155	145	152	178									
	ΔP = SBP3_h_-SBP_0h_	−10	−10	−58	−34	−66									
	**ΔP average**	**−35**^**b**^													
	**SEM**	**12**													
G_AH_		181	184	184	162	159	174	203	199	208	183	162	168	170	195
		184	184	186	159	161	179	192	184	198	173	166	182	181	191
	average	182	177	188	158	164	177	191	173	199	169	160	177	182	185
		182	182	186	160	161	177	195	185	202	175	163	176	178	190
	post intragastric administration														
		145	166	165	145	140	156	167	–	176	146	123	148	150	146
		156	159	166	156	145	158	161	–	177	148	116	141	158	148
		156	168	158	151	146	155	168	–	177	146	129	145	157	139
	average	152	164	163	151	144	156	165	–	177	147	123	145	155	144
	ΔP = SBP3_h_-SBP_0h_	−30	−17	−23	−9	−18	−20	−30	–	−25	−28	−40	−31	−23	−46
	**ΔP average**	**−26**^**b**^													
	**SEM**	**3**													
G_VIKP_		209	223	223	221	205	208	201	201						
		206	222	201	218	208	209	199	203						
		205	219	208	219	208	205	203	206						
	average	207	221	211	219	207	207	201	203						
	post intragastric administration														
		188	193	183	188	200	193	182	179						
		191	192	181	190	201	183	181	177						
		195	199	177	195	205	188	180	185						
	average	191	195	180	191	202	188	181	180						
	ΔP = SBP3_h_-SBP_0h_	−15	−27	−30	−28	−5	−19	−20	−23						
	**ΔP average**	**−21**^**b**^													
	**SEM**	**3**													
G_E_		188	181	187	183	181	178	190	192						
		186	183	182	187	179	180	196	198						
		191	187	202	185	179	177	191	196						
	average	188	184	190	185	180	178	192	195						
	post intragastric administration														
		151	140	142	145	144	142	157	148						
		145	143	142	147	142	141	146	145						
		142	145	149	142	148	145	145	141						
	average	146	143	144	145	145	143	149	145						
	ΔP = SBP3_h_-SBP_0h_	−42	−41	−46	−40	−35	−36	−43	−51						
	**ΔP average**	**−42**^**b**^													
	**SEM**	**2**													
G_E+VIKP_		173	155	153	151	171	169	171							
		175	169	177	174	175	177	175							
		169	171	175	173	173	174	173							
	average	172	165	168	166	173	173	173							
	post intragastric administration														
		132	138	132	138	132	137	135							
		135	136	135	137	134	132	134							
		137	134	136	135	137	135	136							
	average	135	136	134	137	134	135	135							
	ΔP = SBP3_h_-SBP_0h_	−38	−29	−34	−29	−39	−39	−38							
	**ΔP average**	**−35**^**b**^													
	**SEM**	**2**													

Table 2 shows ACE plasma concentration of different groups assayed. This ELISA immunoassay is based on a competitive inhibition. Calibration curve was calculated according to the manufacturer's directions and the values are as follows: y = 0.7089 e ^(−0.001405.X)^ + 0.107 where “y” means OD at 450nm and “x” is ACE sample concentration in μg/ml. It can be observed that G_W_ group presented extremely low values of ACE levels (0.17 ± 0.02 μg/ml), whereas ACE concentration in G_C_ G_A_ G_E_ y G_E+VIKP_ groups were 13.6–25.8 times higher than control group. API, AH and VIKP (G_API_,G_AH_,G_VIKP_ groups respectively) induced an increase in the ACE levels that was 7.6 to 5.3 times higher than control group (1.3 ± 0.2 μg/ml, 0.90 ± 0.3 μg/ml and 1.1 ± 0.3 μg/ml respectively. p < 0.05). The same trend has been observed in studies evaluating synthetic drugs in hypertension treatments [4,5].

**Table 2:** Plasma ACE concentration at the end of the 3 h treatment. Values are presented as mean ± SEM.

Treatment group	Rat	OD_450_ (nm)	μg/ml	Average	SD	SEM
G_W_	1	0.592	0.14	0.15^a^	0.07	0.03
	2	0.579	0.14			
	3	0.761	0.03			
	4	0.464	0.24			
	5	0.536	0.18			
G_A_	1	0.181	0.80	2.08^b^	0.84	0.42
	2	0.081	2.50			
	3	0.077	2.50			
	4	0.076	2.50			
G_C_	1	0.089	2.50	2.50^b^	0	0
	2	0.078	2.50			
	3	0.09	2.50			
G_API_	1	0.134	1.16	1.35^b^	0.52	0.3
	2	0.11	1.94			
	3	0.156	0.95			
G_HA_	1	0.184	0.79	1.16^b^	0.88	0.39
	2	0.116	1.55			
	3	0.095	2.50			
	4	0.438	0.27			
	5	0.215	0.67			
G_VIKP_	1	0.151	0.99	0.87^b^	0.64	0.32
	2	0.227	0.63			
	3	0.539	0.18			
	4	0.113	1.70			
G_E_	1	0.056	2.50	2.50^b^	0	0
	2	0.057	2.50			
	3	0.057	2.50			
	4	0.056	2.50			
	5	0.062	2.50			
	6	0.057	2.50			
	7	0.058	2.50			
G_E+VIKP_	1	0.062	2.50	2.29^b^	0.46	0.2
	2	0.055	2.50			
	3	0.119	1.45			
	4	0.06	2.50			
	5	0.057	2.50			

Table 3 shows renin plasma concentration in the different samples assayed. Calibration curve obtained was y = 0.005x + 0.008 where “y” means OD at 450 nm and “x” represented renin concentration in pg/ml. Only the G_A_, G_API_ and G_AH_ groups presented differences in plasma renin levels, as compared to G_W_ group. No differences were found in the levels of this enzyme between G_C_, G_E_, G_VIKP_ and G_E+VIKP_ groups and the control group G_W_.

**Table 3:** Plasma renin concentration at the end of the 3 h treatment. Values are presented as mean ± SEM.

Treatment group	Rat	OD_450_ (nm)		pg/ml		Average	SD	SEM
G_W_	1	0.279	0.305	54.2	59.4	42.4^a^	9.9	3.1
	2	0.183	0.171	35	32.6			
	3	0.252	0.221	48.8	42.6			
	4	0.223	0.239	43	46.2			
	5	0.158	0.171	30	32.6			
G_A_	1	0.199	0.175	38.2	33.4	34.2^b^	4.7	1.9
	2	0.216	0.16	41.6	30.4			
	3	0.166	0.158	31.6	30			
G_C_	1	0.193	0.178	37	34	35.1^a^	3.4	1.4
	2	0.176	0.159	33.6	30.2			
	3	0.21	0.185	40.4	35.4			
G_API_	1	0.13	0.151	24.4	28.6	34.0^b^	7.3	2.5
	2	0.228	0.222	44	42.8			
	3	0.137	0.177	25.8	33.8			
	4	0.193	0.184	37	35.2			
G_AH_	1	0.157	0.155	29.8	29.4	30.7^b^	4.1	1.9
	2	0.193	0.152	37	28.8			
	3	0.141	0.143	26.6	27			
	4	0.194	0.157	37.2	29.8			
G_VIKP_	1	0.192	0.15	36.8	28.4	34.9^a^	4.7	1.9
	2	0.221	0.17	42.6	32.4			
	3	0.18	0.181	34.4	34.6			
G_E_	1	0.207	0.167	39.8	31.8	35.1^a^	4.7	1.9
	2	0.163	0.159	31	30.2			
	3	0.212	0.192	40.8	36.8			
G_E+VIKP_	1	0.237	0.209	45.8	40.2	36.3^a^	9.6	3
	2	0.192	0.175	36.8	33.4			
	3	0.236	0.227	45.6	43.8			
	4	0.124	0.115	23.2	21.4			

Table 4 shows % ACE activity/μg ACE in plasma collected after treatments. Data are expressed as relative to 100% ACE activity to water control group (G_W_). The lowest activity values (4–7% active ACE/μg ACE) corresponded to groups G_C_, G_A_, G_E_ and G_E+VIKP_, whereas the highest activity values were found in G_VIKP_, G_AH_ and G_API_ groups, which presented 20–13% active ACE/μg ACE. The administration of different samples decreases the enzymatic activity of ACE, together with an increase in plasma levels (Table 2), probably to counter balance the inhibitory effect exerted by the hypotensive peptides.

**Table 4:** Plasma ACE activity. These data are expressed as relative to 100% ACE activity to water control group (Cw). Data are presented as mean ± SEM.

Treatment group	Rats	DO_382_(nm)		μg enzyme	DO_382_/μg enzyme		Activity (%)/μg enzyme		Average	SD	SEM
G_W_	1	0.794	0.773	0.0037	214.6	208.8	104.5	101.7	100.0^a^	4.8	0.01
	2	0.790	0.725	0.0037	213.4	196.0	103.9	95.5			
	3	0.689	0.752	0.0037	186.3	203.3	90.7	99.0			
	4	0.785	0.798	0.0037	212.2	215.8	103.4	105.1			
	5	0.768	0.721	0.0037	207.7	195.0	101.2	95.0			
G_A_	1	0.845	0.771	0.0519	16.3	14.9	7.8	7.1	7.3^b^	0.2	0.01
	2	0.786	0.781	0.0519	15.1	15.0	7.3	7.2			
	3	0.791	0.781	0.0519	15.2	15.0	7.3	7.2			
	4	0.771	0.801	0.0519	14.9	15.4	7.1	7.4			
G_C_	1	0.733	0.790	0.0625	11.7	12.6	5.6	6.1	5.8^b^	0.2	0.01
	2	0.764	0.785	0.0625	12.2	12.6	5.9	6.0			
	3	0.710	0.756	0.0625	11.4	12.1	5.5	5.8			
G_API_	1	0.723	–	0.0338	21.4	–	10.3		10.7^b^	0.3	0.01
	2	0.761	–	0.0338	22.5	–	10.8				
	3	0.757	0.785	0.0338	22.4	23.2	10.8	11.2			
	4	0.748	0.739	0.0338	22.1	21.9	10.6	10.5			
G_AH_	1	0.747	0.756	0.0218	34.3	34.7	16.5	16.7	16.9^b^	0.5	0.01
	2	0.716	0.752	0.0218	32.8	34.5	15.8	16.6			
	3	0.791	0.747	0.0218	36.3	34.3	17.5	16.5			
	4	0.801	0.776	0.0218	36.7	35.6	17.7	17.2			
	5	0.776	0.771	0.0218	35.6	35.4	17.2	17.0			

G_VIKP_	1	0.794	0.799	0.0218	36.4	36.7	17.5	17.6	17.7^b^	0.5	0.01
	2	0.809	0.799	0.0218	37.1	36.7	17.8	17.6			
	3	0.744	0.819	0.0218	34.1	37.6	16.4	18.1			
	4	0.804	0.799	0.0218	36.9	36.7	17.7	17.6			
	5	0.840	0.814	0.0218	38.6	37.3	18.5	17.9			
	6	0.824	0.809	0.0218	37.8	37.1	18.2	17.8			
	7	0.799	0.785	0.0218	36.7	36.0	17.6	17.3			

G_E_	1	0.742	0.765	0.0625	11.9	12.2	5.7	5.9	5.8^b^	0.1	0
	2	0.785	0.790	0.0625	12.6	12.6	6.0	6.1			
	3	0.742	0.756	0.0625	11.9	12.1	5.7	5.8			
	4	0.751	0.756	0.0625	12.0	12.1	5.8	5.8			
	5	0.765	0.770	0.0625	12.2	12.3	5.9	5.9			
	6	0.742	0.747	0.0625	11.9	12.0	5.7	5.8			
	7	0.725	0.775	0.0625	11.6	12.4	5.6	6.0			

G_E+VIKP_	1	0.785	0.684	0.0573	13.7	11.9	6.6	5.7	6.4^b^	0.3	0.01
	2	0.770	0.785	0.0573	13.4	13.7	6.5	6.6			
	3	0.775	0.761	0.0573	13.5	13.3	6.5	6.4			
	4	0.756	0.765	0.0573	13.2	13.4	6.4	6.4			
	5	0.821	0.765	0.0573	14.3	13.4	6.9	6.4			

Table 5 shows the contractile activity determined in presence of potassium ions. Contractile force was higher in G_W_, G_C_ and G_A_ groups (roughly 0.45 g/mg), whereas this activity was significantly lower in the animals belonging to groups G_API_, G_VIKP_, G_E+VIKP_, G_AH_ and G_E_ (0.34–0.29 g/mg). Upon treating aorta rings with physiological concentrations of norepinephrine, statistically differences were observed in G_VIKP_ and G_E+VIKP_ groups.

**Table 5:** Effect of different samples on isolated aortic rings contracted by exposure to a high concentration of: Potassium ion (80 mM) and Norepinephrine (10^−6^ M). F_b1_ (g): basal force before K addition. F_b2_ (g): basal force before N addition. F_c_ (g): contractile force. N: norepinephrine. K: potassium ion. AW: aorta weight. Data are presented as mean ± SEM.

Treatment group	Rats	F_b1_ (g)	F_c_ K (g)	F_b2_ (g)	F_c_ N (g)	AW (mg)	F_c_ K (g/mg)	F_c_ N (g/mg)
G_w_	1	0	1.15	0.18	2.06	2.69	0.428	0.699
	2	0.07	1.28	0.22	1.79	2.33	0.519	0.674
	3	−0.08	0.8	0.14	1.2	2.24	0.393	0.473
	4	−0.13	1.08	0.12	1.25	3.4	0.356	0.332
	5	−0.03	1.18	0.02	1.09	3.21	0.377	0.333
	6	0.02	1.2	0.14	1.36	2.58	0.457	0.473
	average						**0.42**^**a**^	**0.50**^**a**^
	SD						**0.06**	**0.17**
	SEM						**0.02**	**0.07**
G_A_	1	−0.042	1.113	0.056	1.253	3.47	0.333	0.345
	2	−0.07	1.015	−0.007	1.239	2.00	0.543	0.623
	3	0.021	1.113	0.0035	1.253	2.42	0.451	0.516
	4	0.021	0.742	0.049	1.001	1.95	0.370	0.538
	average						**0.44**^**a**^	**0.49**^**a**^
	SD						**0.11**	**0.14**
	SEM						**0.05**	**0.07**
G_C_	1	0.03	1.68	−0.15	1.29	3.95	0.418	0.365
	2	0.07	1.54	0.59	1.85	3.45	0.426	0.365
	3	−0.05	1.34	0	1.57	3.12	0.446	0.503
	average						**0.43**^**a**^	**0.41**^**a**^
	SD						**0.01**	**0.08**
	SEM						**0.01**	**0.05**
G_API_	1	2.1	2.95	2.1	3.48	2.56	0.332	0.539
	2	1.98	2.67	2.19	3.21	3.12	0.221	0.327
	3	2.08	3.29	2.29	3.73	3.94	0.307	0.365
	4	0.1	0.9	0.3	1.2	2.85	0.281	0.316
	5	0	0.9	0.1	1.1	1.9	0.474	0.526
	6	0	1	0.2	1.3	2.63	0.380	0.418
	average						**0.332**^**b**^	**0.415**^**a**^
	SD						**0.087**	**0.098**
	SEM						**0.036**	**0.040**
G_AH_	1	−0.1	1.2	0.4	1.5	2.50	0.52	0.44
	2	−0.1	1.5	0.3	1.8	2.98	0.537	0.503
	3	0	0.9	0.2	1.1	2.38	0.378	0.378
	4	−0.02	0.5995	−0.275	0.8085	2.06	0.301	0.526
	5	0.06	0.6655	−0.06	0.9075	1.90	0.319	0.509
	6	−0.04	0.8525	−0.099	1.089	1.91	0.467	0.622
	7	0.07	0.528	−0.165	0.759	2.24	0.204	0.413
	8	0.03	0.7645	−0.0495	0.935	2.20	0.334	0.448
	9	−0.04	0.72	−0.126	0.894	2.22	0.342	0.459
	10	−0.01	0.8262	0.0108	10.476	2.41	0.347	0.430
	11	0.04	0.624	0.042	0.72	2.01	0.291	0.337
	12	−0.07	0.402	−0.042	0.528	1.87	0.252	0.305
	13	0.02	0.520	0.016	0.668	3.28	0.152	0.199
	average						**0.340**^**b**^	**0.43**^**a**^
	SD						**0.11**	**0.11**
	SEM						**0.03**	**0.03**
G_VIKP_	1	0.014	0.868	0.049	1.316	3.15	0.271	0.402
	2	0.077	0.861	0.077	1.239	3.00	0.261	0.387
	3	−0.098	0.84	−0.049	1.204	3.30	0.284	0.380
	4	0.014	1.162	0.07	1.61	2.86	0.401	0.538
	5	0.02	1.27	−0.15	0.84	2.75	0.455	0.360
	6	0	1.056	0	1.04	2.81	0.376	0.370
	7	0	1.24	−0.016	1.1	3.37	0.368	0.331
	8	0.056	1.008	−0.04	0.9	3.61	0.264	0.260
	average						**0.335**^**b**^	**0.379**^**b**^
	SD						**0.069**	**0.073**
	SEM						**0.03**	**0.03**
G_E_	1	0.04	0.979	−0.0825	1.094	2.42	0.388	0.486
	2	−0.02	0.715	−0.044	0.902	1.61	0.457	0.588
	3	0.0495	0.891	0	1.144	2.02	0.417	0.566
	4	0.0162	0.6696	−0.1134	0.8748	2.30	0.284	0.430
	5	0.1	0.476	−0.413	0.826	2.85	0.132	0.435
	6	0.24	0.567	−0.287	0.623	2.00	0.164	0.455
	7	0.26	0.616	0.056	1.008	2.09	0.170	0.456
	average						**0.287**^**b**^	**0.488**^**a**^
	SD						**0.135**	**0.064**
	SEM						**0.05**	**0.02**
G_E+VIKP_	1	−0.012	0.69	−0.06	0.768	2.73	0.257	0.303
	2	0.0616	0.6496	−0.028	0.6216	2.44	0.241	0.266
	3	−0.03	0.78	−0.115	0.855	2.79	0.290	0.348
	4	0.028	0.8008	−0.056	0.9968	2.60	0.297	0.405
	5	0.02	1.2	−0.11	0.75	2.70	0.437	0.319
	6	−0.01	1.05	−0.11	0.642	3.13	0.339	0.240
	7	−0.08	1.1	−0.17	0.602	3.01	0.392	0.256
	average						**0.322**^**b**^	**0.305**^**b**^
	SD						**0.072**	**0.058**
	SEM						**0.03**	**0.02**

## 2 Experimental design. Materials and methods

### 2.1 Samples

The following samples were used for *in vivo* assays:

- Amaranth protein isolate (API) and hydrolysate (AH) prepared from *Amaranthus hypochondriacus* as described elsewhere [2]. The protein content was 87 ± 1 and 57 ± 2% w/w w.b. for API and AH respectively.
- VIKP peptide, which is a synthetic peptide from 11S amaranth protein. This peptide has inhibitory activity on ACE [6].
- O/W 20:80 emulsions prepared with sunflower oil and 1:1 protein mixture of API and AH at pH 2 with a total protein concentration of 2% w/v with or without VIKP peptide [(API50 + AH50)-2%+VIKP and (API50 + AH50)-2%, respectively].

Emulsions were prepared according to [2]. Emulsions were frozen at −80 °C, lyophilized and resuspended as required. Before administration, the resuspended emulsions were homogenized with a magnetic stirring bar.

- Commercial ACE and renin inhibitors (captopril and aliskiren, respectively) were employed as positive controls.

### 2.2 *In vivo* assays

#### 2.2.1 Indirect measurement of blood pressure

The systolic blood pressure was measured according to [3]. In order to determine baseline values, blood pressure values were recorded at least three times on different days for each rat. After recording the last baseline blood pressure value, an aqueous suspension of each sample was administered to each animal. Three hours after the administration, blood pressure values were recorded with a tail cuff and a pulse sensor (NarcoBiosystems, Houston, TX).

#### 2.2.2 Determination of plasma ACE and renin concentrations

A commercial ELISA kit (Rat Angiotensin converting enzyme MBS703086, MyBioSource, CA, USA) was employed to determine ACE concentration according to manufacturer's directions. This immunoassay is based on a competitive inhibition. Briefly, microtitre plates are coated with ACE. Samples and standards are incubated together with an anti-ACE HRP-labeled conjugate to generate the competition. Plasma renin concentration was determined with a commercial ELISA kit (Rat renin ELISA kit MBS041519 MyBioSource) following the manufacturer's directions. This immunoassay is a direct ELISA, which has an analytical measurement range of 6.25–200 pg/ml. The final colour reaction was read in a microtiter plate reader (Biotek Synergy HT, Winooski, VT, USA) at 450 nm.

#### 2.2.3 Determination of plasma ACE activities

ACE-inhibitory activity was assayed according to [2]. Briefly, to determine the enzymatic activity, 50 μl of buffer [0.2M sodium borate pH 8.3; 2M NaCl), 25 μl of milli Q water, 25 μl of the commercial enzyme (maximum activity control)], or plasma samples were incubated with 100 μl of synthetic substrate (HHL) at 37 °C for 30 min. The reaction was stopped by heating the mixture over a water bath at 90 °C for 15 min. After cooling, 600 μl of 0.2M potassium pH 8.2 and 515 μl of colour reagent, which reacts with the hippuric acid generated during the enzymatic reaction, were added and stirred vigorously with a vortex and then centrifuged for 10 min at 20 °C and 3000×*g*. The absorbance was measured at 382 nm in a spectrophotometer (Beckman DU 650). The reaction blank was obtained by incubating the synthetic substrate with neither the plasma samples nor the enzyme, completing the reaction volume with milli Q water. Reaction blanks without the substrate (HHL was replaced by 100 μl of borate buffer) and containing plasma samples were also included. Controls containing plasma samples and captopril were also assayed.

#### 2.2.4 *Ex vivo* experiments

During the surgical procedure employed to obtain blood samples, the thoracic aorta was resected and placed in saline solution bubbled with 5% CO_2_ and 95% O_2_. The adjacent connective tissue was carefully removed avoiding distention of the vessel and damage to the endothelium. The aorta was then cut into 2 mm long rings. Assay was performed according to [3]. The rings were gently suspended between two stainless steel wires in a water-jacketed organ baths kept at 37 °C and filled with saline solution, bubbled with a mixture of 5% CO_2_ and 95% O_2_, giving a pH of 7.40. The lower wire was fixed to a vertical plastic rod immersed in the organ bath, while the upper one was rigidly connected to a force transducer (Grass FT.03D, Grass Telefactor, West Warwick, CT, USA). Preparations were then stretched to obtain a passive force of 2 g and stabilized during 1 h, changing the solution in the chamber every 20 min. Tissue rings were then exposed to a solution containing 80mM potassium or norepinephrine 10^−6^ M. For each condition, the contractile response was recorded. At the end of the experiment, tissue rings were dried on filter paper and weighed on a precision scale. The contraction intensity was calculated as the quotient between strength and the weight of the ring (mgF/mg).
